# Detection of miRNA in chronic spontaneous urticaria patients - pilot study

**DOI:** 10.3389/fimmu.2026.1865847

**Published:** 2026-07-29

**Authors:** Lāsma Lapiņa, Katrīna Daila Neiburga-Vīgante, Linda Gailīte, Dmitrijs Rots, Nataļja Kurjāne

**Affiliations:** 1 Institute of Oncology and Molecular Genetics, Riga Stradiņš University, Riga, Latvia; 2 Centre for Clinical Immunology and Allergology, Pauls Stradiņš Clinical University Hospital, Riga, Latvia; 3 Centre for Diagnosis and Treatment of Allergic Diseases, Riga, Latvia; 4 University of Tartu, Tartu, Estonia; 5 Children's Clinical University Hospital, Riga, Latvia

**Keywords:** angioedema, biomarker discovery, chronic spontaneous urticaria, immune-mediated inflammation, MicroRNAs, miRNA profiling, molecular endotyping

## Abstract

**Background:**

Chronic spontaneous urticaria (CSU) is a heterogeneous immune-mediated disorder characterized by recurrent wheals and/or angioedema. Despite advances in understanding its pathogenesis, robust biomarkers for disease stratification remain lacking. MicroRNAs (miRNAs) are key post-transcriptional regulators implicated in immune-mediated diseases and may provide novel insights into CSU.

**Objective:**

To characterize circulating miRNA expression profiles in CSU and explore their association with clinical phenotypes.

**Methods:**

Patients were stratified into three groups: urticaria only (n=10), urticaria with angioedema (n=10), and angioedema only (n=10), alongside healthy controls (n=10). Plasma miRNAs were sequenced using the NovaSeq 6000 platform. Data were processed with the nf-core smrnaseq pipeline, followed by multivariate and differential expression analyses in R. Functional enrichment and target prediction analyses were performed using KEGG, Reactome, and WikiPathways.

**Results:**

Thirty patients (mean age 45.3 years; 76.7% female) were included. Overall, 61 miRNAs were differentially expressed (p < 0.05) across groups, including controls. No miRNAs remained significant after multiple testing correction in pairwise comparisons between clinical subgroups. However, several miRNAs (miR-204-5p, miR-3158-3p, miR-4732-3p, miR-576-5p, and miR-877-5p) showed nominal associations with disease phenotypes. miR-204-5p demonstrated a trend toward reduced expression in patients with urticaria and angioedema (adjusted p = 0.05).

**Conclusion:**

Circulating miRNA profiles may reflect biological heterogeneity in CSU. Although no subgroup-specific signatures were confirmed after correction for multiple testing, several candidate miRNAs were identified, supporting further investigation of miRNA-based biomarkers in CSU.

## Introduction

Chronic spontaneous urticaria (CSU) is a complex immune-mediated inflammatory skin disorder characterized by the spontaneous and recurrent occurrence of wheals and/or angioedema lasting for more than six weeks in the absence of identifiable external triggers (1–4). The disease is driven by dysregulated activation of mast cells and basophils, leading to the release of histamine and a range of pro-inflammatory mediators that promote vasodilatation, increased vascular permeability, and sensory nerve activation underlying the characteristic symptoms (2, 5, 6).

Approximately half of CSU patients show evidence of autoimmune processes, whereas the remainder exhibit non-autoimmune immune dysregulation (2, 4, 7, 8). Chronic inflammation is sustained by a complex cytokine and chemokine network involving both innate and adaptive immune pathways (9–13), together with additional non–IgE-mediated mechanisms contributing to disease pathophysiology (2, 14–20).

Within this immunopathological context, microRNAs (miRNAs) have emerged as important post-transcriptional regulators with potential relevance to CSU (21, 22). miRNAs are short, non-coding RNA molecules that modulate gene expression through sequence-specific interactions with target messenger RNAs, thereby influencing immune cell differentiation, activation, cytokine production, and inflammatory signaling pathways (21, 23). Dysregulated miRNA expression has been implicated in chronic inflammation and autoimmune responses across multiple immune-mediated diseases (22, 24).

In CSU, evidence suggests that altered miRNA profiles may regulate key disease-related processes, including mast cell activation, FcϵRI signaling, IgE production, cytokine networks, and endothelial function (25, 26). Previous studies have provided initial evidence for the involvement of specific miRNAs in chronic urticaria. Qu et al. showed that miR-194 may reduce inflammatory responses and human dermal microvascular endothelial cell permeability in CSU by targeting THBS1 and suppressing the TGF-β/SMAD pathway (27). Lin et al. identified 16 differentially expressed miRNAs in patients with CSU and active hives, including several miRNAs associated with a positive CU index, suggesting their potential relevance as biomarkers of autoimmune urticaria (28). In addition, miRNA dysregulation has been increasingly recognized across immune-mediated and inflammatory dermatological diseases, supporting the broader relevance of miRNA-based research in dermatology (29). However, current data remain limited, and the relationship between specific miRNA expression patterns and distinct clinical phenotypes of CSU is not fully understood.

Given the marked clinical and immunological heterogeneity of CSU, miRNAs represent promising candidate biomarkers for disease stratification and improved understanding of disease mechanisms. The aim of this study was to characterize miRNA expression profiles in patients with CSU and to evaluate their association with disease presence and distinct clinical phenotypes.

## Methods

### Patients

The study cohort was identified using electronic medical records of Pauls Stradiņš Clinical University Hospital and included adult patients diagnosed with CSU between January 2016 and May 2023. Additional participants were recruited through the Centre for Diagnosis and Treatment of Allergic Diseases.

CSU diagnosis was established according to the EAACI/GA²LEN/EuroGuiDerm/APAAACI guidelines, defined as the presence of spontaneous wheals, angioedema, or both persisting for more than six weeks in the absence of identifiable external triggers (1). All diagnoses were confirmed by a board-certified allergologist.

The study was approved by the Ethics Committee of Riga Stradiņš University (Approval No. 2-PĒK-4/68/2023), and all participants provided written informed consent.

From 207 adult CSU patients invited to participate, 30 were randomly selected for inclusion in the miRNA analysis and assigned to predefined clinical phenotype groups: wheals only (HSN), wheals with angioedema (HSNA), and angioedema without wheals (IA), with 10 patients in each group. miRNA expression was analysed in these 30 patients (mean age 45.3 years, SD ±14.3), of whom 76.7% (n=23) were women (CSU patient cohort described in **Table 1**).

**Table 1:** Demographic, clinical, and laboratory characteristics of patients with CSU in the study cohort.

Variable	HSN (n=10)	HSNA (n=10)	IA (n=10)	Overall (n=30)
Sample size, n	10	10	10	30
Age, years (Mean ± SD)	45.9 ± 9.3 (n=10)	38.0 ± 16.4 (n=10)	51.9 ± 14.0 (n=10)	45.3 ± 14.3 (n=30)
Female sex, n (%)	7/10 (70%)	7/10 (70%)	9/10 (90%)	23/30 (77%)
BMI, kg/m² (Mean ± SD)	25.5 ± 5.8 (n=10)	25.7 ± 5.7 (n=10)	27.9 ± 5.5 (n=10)	26.3 ± 5.6 (n=30)
Urban residence, n (%)	9/10 (90%)	7/10 (70%)	9/10 (90%)	25/30 (83%)
Disease duration, months (Median [IQR])	36.0 [12.0; 60.0] (n=10)	45.0 [15.0; 96.0] (n=10)	45.0 [30.0; 72.0] (n=10)	45.0 [18.0; 72.0] (n=30)
VAS (Mean ± SD)	7.6 ± 1.6 (n=10)	7.7 ± 1.4 (n=10)	NA	7.7 ± 1.5 (n=20)
UCT (Mean ± SD)	3.9 ± 3.0 (n=10)	4.7 ± 2.3 (n=10)	NA	4.3 ± 2.6 (n=20)
UAS7 (Mean ± SD)	27.0 ± 15.4 (n=10)	30.8 ± 7.4 (n=10)	NA	28.9 ± 11.9 (n=20)
CU-Q2oL (Mean ± SD)	43.4 ± 22.8 (n=10)	54.8 ± 14.9 (n=10)	NA	49.1 ± 19.6 (n=20)
AAS7 (Mean ± SD)	NA	NA	50.8 ± 42.6 (n=10)	50.8 ± 42.6 (n=10)
AECT-3mo (Mean ± SD)	NA	NA	5.0 ± 3.6 (n=10)	5.0 ± 3.6 (n=10)
AE-QoL (Mean ± SD)	NA	NA	58.2 ± 28.9 (n=10)	58.2 ± 28.9 (n=10)
Currently receiving any listed therapy, n (%)	8/10 (80%)	9/10 (90%)	7/10 (70%)	24/30 (80%)
Antihistamine, n (%)	3/10 (30%)	5/10 (50%)	4/10 (40%)	12/30 (40%)
Omalizumab, n (%)	0/10 (0%)	2/10 (20%)	1/10 (10%)	3/30 (10%)
Total IgE, IU/mL (Median [IQR])	117.0 [31.4; 295.8] (n=10)	98.7 [58.2; 192.5] (n=10)	89.0 [54.0; 154.0] (n=9)	92.0 [53.5; 193.0] (n=29)
CRP, mg/L (Median [IQR])	4.0 [4.0; 4.0] (n=10)	4.0 [4.0; 4.0] (n=10)	4.0 [4.0; 4.0] (n=10)	4.0 [4.0; 4.0] (n=30)
D-dimer, mg/L (Median [IQR])	0.3 [0.2; 0.3] (n=5)	0.3 [0.2; 1.3] (n=6)	0.4 [0.3; 1.7] (n=8)	0.3 [0.2; 0.5] (n=19)
Anti-TPO positive, n (%)	1/6 (17%)	4/6 (67%)	3/5 (60%)	8/17 (47%)
Any allergy history, n (%)	6/10 (60.0%)	5/10 (50.0%)	4/10 (40.0%)	15/30 (50.0%)
Any comorbidity, n (%)	8/10 (80.0%)	5/10 (50.0%)	10/10 (100.0%)	23/30 (76.7%)
Autoimmune disease, n (%)	1/10 (10.0%)	2/10 (20.0%)	6/10 (60.0%)	9/30 (30.0%)
Thyroid/endocrine disease, n (%)	3/10 (30.0%)	3/10 (30.0%)	0/10 (0.0%)	6/30 (20.0%)
Gastrointestinal disease, n (%)	2/10 (20.0%)	3/10 (30.0%)	5/10 (50.0%)	10/30 (33.3%)
Cardiovascular disease, n (%)	1/10 (10.0%)	0/10 (0.0%)	4/10 (40.0%)	5/30 (16.7%)
Respiratory disease, n (%)	3/10 (30.0%)	0/10 (0.0%)	3/10 (30.0%)	6/30 (20.0%)
Psychiatric/neurological disease, n (%)	1/10 (10.0%)	0/10 (0.0%)	2/10 (20.0%)	3/30 (10.0%)
Oncological disease, n (%)	0/10 (0.0%)	0/10 (0.0%)	1/10 (10.0%)	1/30 (3.3%)
Smoking and/or alcohol exposure, n (%)	6/10 (60.0%)	6/10 (60.0%)	3/10 (30.0%)	15/30 (50.0%)

Data are presented as mean ± SD, median [IQR], or n (%). NA indicates not applicable or not assessed. HSN, wheals without angioedema; HSNA, wheals with angioedema; IA, angioedema without wheals; CSU, chronic spontaneous urticaria; VAS, visual analogue scale; UCT, urticaria control test; UAS7, urticaria activity score over 7 days; CU-Q2oL, chronic urticaria quality of life questionnaire.

A control group of 10 healthy adult individuals without a history of CSU, allergic disease, or other chronic inflammatory or autoimmune conditions was included. Control participants were selected to approximate the patient cohort with respect to age and sex distribution.

### Clinical assessment and sample collection

All participants underwent a structured clinical evaluation by a board-certified allergologist. Standardized questionnaires were used to collect data on medical history, comorbidities, ongoing therapies, and disease-specific characteristics, including symptom type, anatomical distribution, duration, and intensity.

Peripheral venous blood samples were collected in EDTA tubes under standard conditions. Plasma was obtained by centrifugation at 1500 × g for 10 minutes, aliquoted, and stored at −80 °C until further analysis. Sample collection was performed between October 2022 and May 2023.

### miRNA sequencing

Circulating miRNAs were isolated from plasma using standardized protocols to preserve RNA integrity. miRNA isolation, library preparation, and sequencing were performed by CeGaT GmbH, Tübingen, Germany, according to the laboratory’s standard small RNA sequencing workflow.

Small RNA libraries were prepared using the NEXTFLEX Small RNA-Seq v3 library preparation kit (PerkinElmer) and sequenced on the Illumina NovaSeq 6000 platform, generating 50-bp single-end reads. As miRNA expression was assessed by small RNA sequencing rather than target-specific RT-qPCR, no miRNA-specific primers or hydrolysis probes were used.

According to the manufacturer’s protocol, the NEXTFLEX Small RNA-Seq v3 workflow includes adapter ligation, reverse transcription, PCR amplification using indexed barcode primers, and size selection of small RNA libraries. Adapter trimming and removal of low-quality reads were performed during downstream bioinformatic processing.

Raw sequencing data were processed using the nf-core/smrnaseq pipeline, including adapter trimming, quality filtering, alignment to the human reference genome GRCh38, and miRNA quantification against annotated human miRNA references. Reads with a Phred quality score <30 or shorter than 15 nucleotides after trimming were excluded from downstream analyses.

### Statistical analysis

miRNA counts were normalized using the trimmed mean of M-values (TMM) method. Differences in miRNA expression between groups were assessed using multivariate 50–50 MANOVA. Differential expression analysis was performed in R (version 4.3.0) using the limma-voom framework with empirical Bayes moderation.

P-values were adjusted for multiple testing using the Benjamini–Hochberg false discovery rate (FDR), with FDR <0.05 considered statistically significant.

To explore the biological relevance of dysregulated miRNAs, target prediction and pathway enrichment analyses were conducted using KEGG, Reactome, and WikiPathways via the MIENTURNET web platform.

## Results

### Differential miRNA expression between controls and CSU patients

Differential expression analysis comparing healthy controls with all CSU patients identified 45 miRNAs with significantly altered expression. Of these, 18 miRNAs met the predefined prioritization criteria (false discovery rate [FDR] < 0.05, |log_2_ fold change [log_2_FC]| > 2, and mean expression > 5) and were selected for downstream analyses.

Several miRNAs were markedly downregulated in CSU patients, including miR-485-3p, miR-483-3p, miR-486-3p, miR-664a-3p, miR-1180-3p, and let-7b-5p. In contrast, miR-21-5p, miR-29a-3p, miR-148a-3p, miR-335-5p, miR-144-3p, and miR-215-5p were significantly upregulated. Notably, let-7b-5p and miR-21-5p exhibited both large absolute fold changes and strong statistical significance, indicating robust differences between CSU patients and controls (**Table 2**).

**Table 2:** Differentially expressed miRNAs between controls and CSU patients.

miRNA	log2FC	Average expression	Adjusted p-value (FDR)
miR-485-3p	-3.29	5.87	1.88E-02
miR-483-3p	-2.72	5.71	3.80E-02
miR-664a-3p	-2.67	5.07	2.79E-02
miR-486-3p	-2.62	8.58	2.28E-05
miR-1180-3p	-2.52	5.41	3.50E-02
let-7b-5p	-2.50	15.18	5.71E-15
miR-454-3p	-2.45	5.90	4.11E-02
miR-744-5p	-2.42	7.46	5.82E-03
miR-3173-5p	-2.19	6.15	2.42E-02
miR-142-3p	2.00	6.10	3.97E-02
miR-21-5p	2.02	14.67	5.51E-15
miR-29a-3p	2.11	10.47	1.00E-11
miR-148a-3p	2.14	11.12	9.20E-12
miR-340-5p	2.35	7.64	1.31E-03
miR-144-3p	2.43	8.29	5.13E-05
miR-29c-3p	2.53	7.38	6.90E-04
miR-335-5p	2.75	10.22	3.29E-12
miR-215-5p	4.15	5.90	1.73E-04

miRNA, microRNA; log_2_FC, log_2_ fold change; FDR, false discovery rate.

### miRNA expression patterns across diagnostic groups

To further evaluate miRNA expression differences across diagnostic groups (Control, HSN, HSNA, IA), a multi-group 50–50 MANOVA identified 61 significantly altered miRNAs (FDR < 0.05).

**Figure 1:**
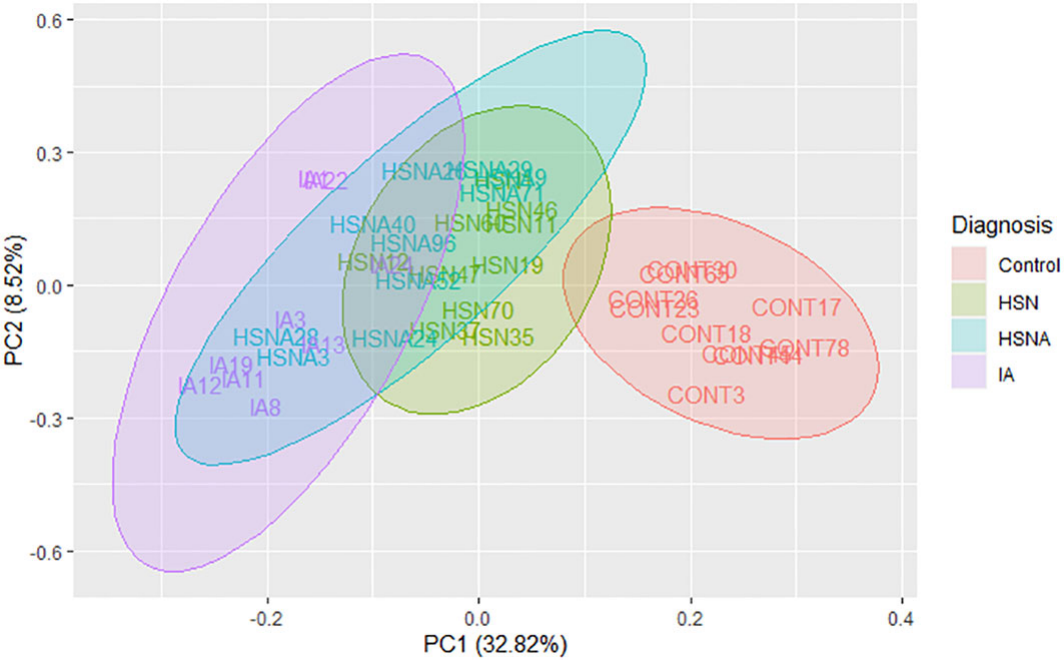
Principal component analysis of miRNA expression across diagnostic groups. Principal component analysis (PCA) of miRNA expression profiles across healthy controls and CSU patient subgroups (HSN, HSNA, IA). PC1 and PC2 explain 32.82% and 8.52% of the total variance. Controls form a distinct cluster, whereas CSU subgroups show substantial overlap. PCA, principal component analysis; HSN, wheals only; HSNA, wheals with angioedema; IA, angioedema only.

Principal component analysis (PCA) based on these miRNAs demonstrated a clear separation between controls and CSU patients, whereas substantial overlap was observed among CSU subgroups, indicating limited molecular discrimination between clinical phenotypes (**Figure 1**).

### Target gene interaction analysis of significant miRNAs

**Figure 2:**
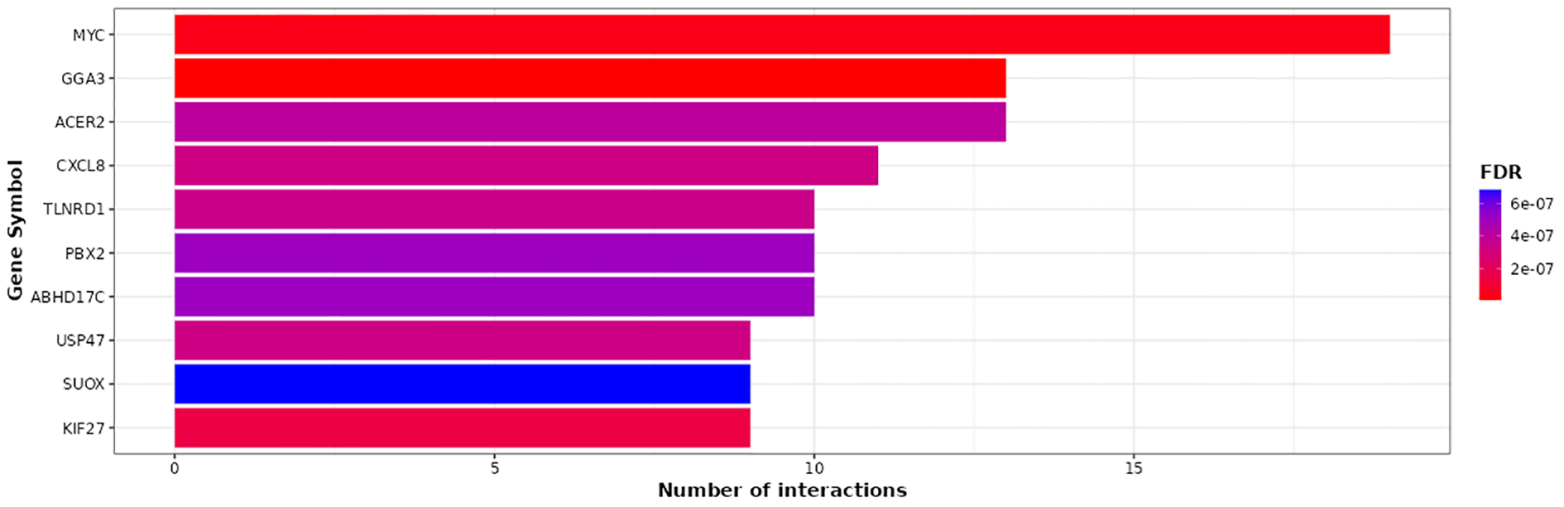
Highly connected miRNA target genes identified by multi-group analysis. Bar plot showing genes with the highest number of interactions with significantly altered miRNAs. Bar height represents interaction counts, and color indicates the false discovery rate (FDR).

To investigate the regulatory relevance of the identified miRNAs, predicted target genes were analyzed using MIENTURNET. Ranking targets by interaction frequency revealed several highly connected genes, including *MYC*, *GGA3*, *ACER2*, *CXCL8*, *TLRND1*, *PBX2*, *ABHD17C*, *USP47*, *SUOX*, and *KIF27*. Among these, *MYC* showed the highest number of interactions, suggesting a central role within the inferred miRNA–gene regulatory network (**Figure 2**).

**Figure 3:**
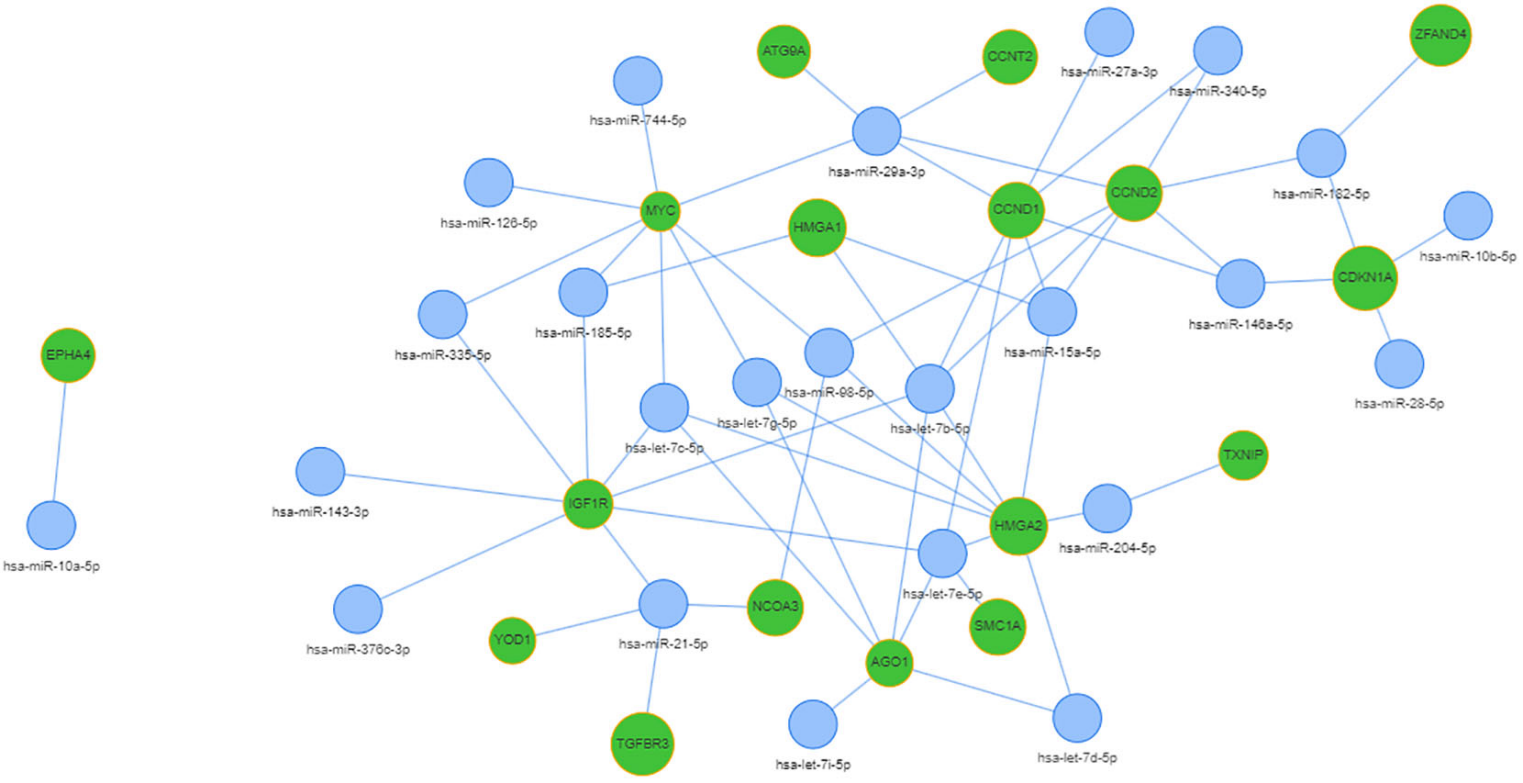
High-confidence miRNA–target interaction network. Interaction network constructed from significantly altered miRNAs, including only strong-evidence interactions and target genes regulated by multiple miRNAs. Blue nodes represent miRNAs, and green nodes represent target genes.

A high-confidence interaction network was subsequently constructed by retaining only strong-evidence interactions and genes targeted by multiple miRNAs. This network highlighted shared regulatory hubs, including *MYC* and *IGF1R*, reflecting coordinated post-transcriptional regulation (**Figure 3**).

### Functional pathway enrichment of miRNA targets

**Figure 4:**
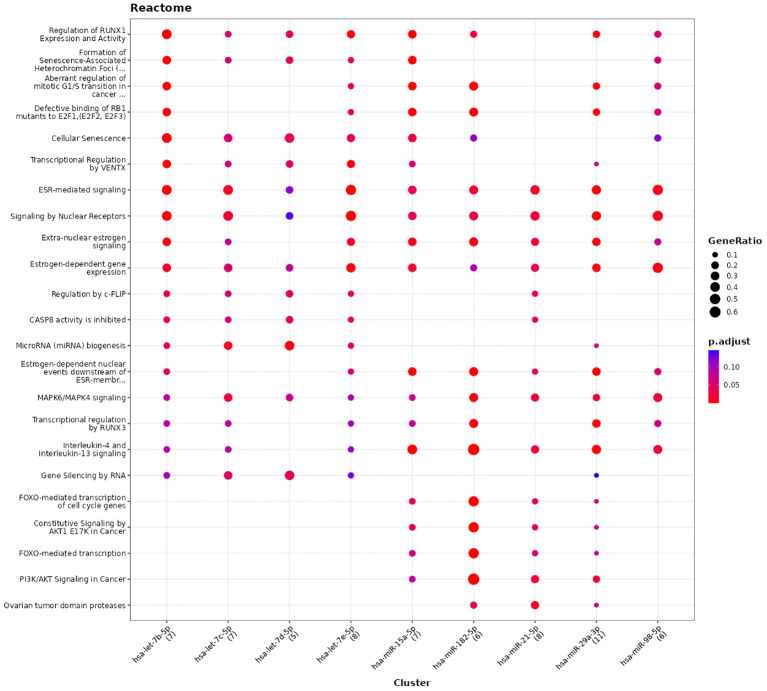
Reactome pathway enrichment of miRNA target genes. Dot plot representing Reactome pathway enrichment analysis. Dot size indicates gene ratio, and color reflects adjusted p-values.

Functional enrichment analysis of target genes associated with highly connected miRNAs revealed significant overrepresentation of pathways related to transcriptional regulation, cell cycle control, and signal transduction. Reactome pathway analysis highlighted TP53- and RUNX1-mediated transcriptional regulation, apoptotic signaling, MAPK signaling, nuclear receptor–associated pathways, and cellular senescence (**Figure 4**).

### Nominal subgroup-associated miRNAs

Pairwise and one-versus-all subgroup comparisons did not identify miRNAs that remained significant after multiple testing correction. However, several miRNAs demonstrated nominal significance (p < 0.05). Five of these overlapped with the multi-group MANOVA results and were considered candidate subgroup-associated markers.

**Figure 5:**
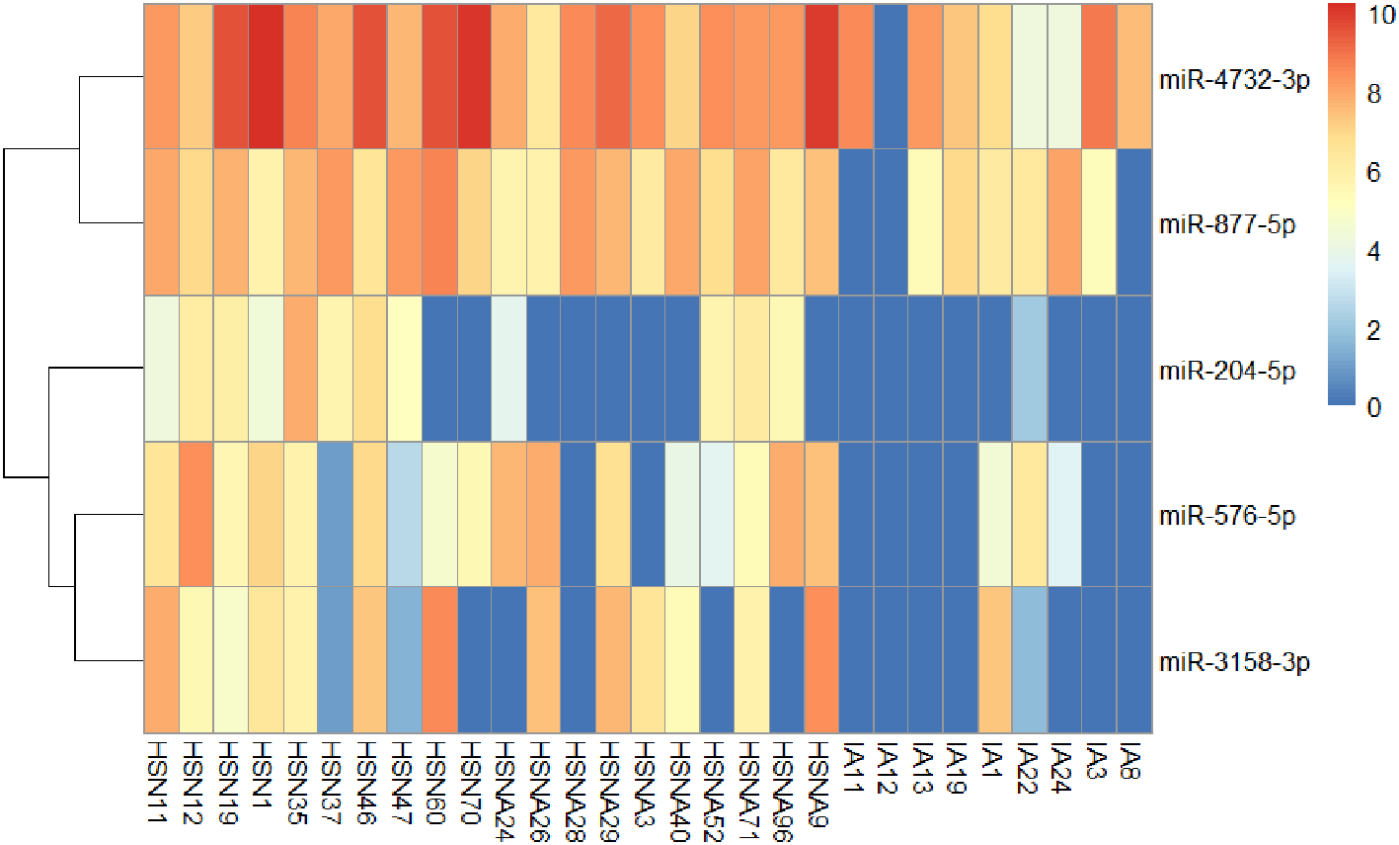
Heatmap of nominally significant miRNAs. Heatmap showing expression patterns of five nominally significant miRNAs (miR-204-5p, miR-3158-3p, miR-4732-3p, miR-576-5p, and miR-877-5p). Samples are grouped by clinical phenotype, and rows are hierarchically clustered.

Visualization of their expression patterns revealed subgroup-related trends alongside substantial within-group heterogeneity (**Figure 5**).

### Hierarchical clustering

**Figure 6:**
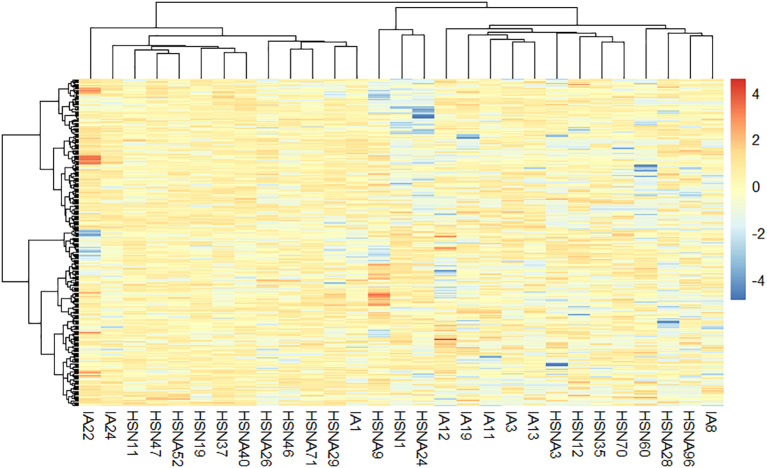
Hierarchical clustering of all samples. Unsupervised hierarchical clustering of miRNA expression profiles across all samples. Clustering separates control and CSU samples and reveals multiple patient clusters with mixed clinical labels.

Unsupervised hierarchical clustering across all samples separated CSU patients from controls and revealed three patient clusters containing mixed clinical phenotypes, indicating molecular heterogeneity beyond predefined clinical categories (**Figure 6**).

In summary, miRNA expression profiling revealed widespread alterations in CSU compared with healthy controls, accompanied by coordinated regulatory patterns involving shared target genes and signaling pathways. While miRNA profiles clearly distinguished CSU patients from controls, separation among clinical subgroups remained limited, reflecting substantial molecular heterogeneity.

## Discussion

This study investigated circulating miRNA expression patterns in healthy controls and clinically defined CSU subgroups (HSN, HSNA, IA) using complementary statistical and functional approaches. The results demonstrate a consistent signal of altered miRNA expression at the case–control level, linked to regulatory programs related to immune signaling, transcriptional control, and cellular stress responses. Separation between closely related clinical subgroups remained limited. Therefore, the main finding of this study is not the identification of a single subgroup-specific miRNA signature, but rather the observation that CSU patients show a broader circulating miRNA dysregulation compared with healthy controls, whereas predefined clinical phenotypes show only modest molecular separation.

Across case–control and multi-group analyses, miRNA dysregulation was consistently observed in CSU patients. Functional enrichment analyses identified pathways related to transcriptional regulation, RNA metabolism, and signal transduction, including PI3K–Akt, MAPK-related signaling, p53/FoxO-associated programs, and apoptosis- and senescence-related processes. These pathways are central to immune activation, stress adaptation, and cell survival. Their identification across KEGG, Reactome, and WikiPathways supports the involvement of miRNA-mediated network-level regulation rather than isolated miRNA–target effects in chronic inflammatory conditions (30, 31). These pathways should not be interpreted as evidence for a single dominant disease mechanism, but rather as indicators of distributed regulatory changes affecting several immune and stress-response networks relevant to chronic inflammation. The observed circulating miRNA expression patterns may contribute to CSU pathobiology through several non-mutually exclusive mechanisms. Altered miRNAs may influence immune activation thresholds by regulating signaling pathways involved in mast cell activation, cytokine responses, and inflammatory mediator release. Enrichment of PI3K–Akt and MAPK-related pathways suggests potential effects on cell activation, survival, and stress-response programs, which are relevant to persistent inflammatory activity. miRNA-related regulation of endothelial function and vascular permeability may be particularly relevant in patients with angioedema, as vascular leakage is a key clinical feature of this phenotype. The convergence of multiple dysregulated miRNAs on shared target networks supports the concept that CSU-associated miRNA dysregulation may act through coordinated post-transcriptional regulation rather than through single miRNA–single target mechanisms.

These findings align with previous studies in chronic inflammatory and autoimmune diseases, where miRNAs modulate immune activation thresholds, cytokine signaling, and cell fate decisions (30–32). In CSU, existing evidence links miRNAs to mast cell activation, FcϵRI signaling, and inflammatory mediator release, supporting their role in disease-relevant immune pathways (26, 33, 34). Recent dermatology-focused reviews have highlighted miRNAs as emerging regulators, biomarkers, and potential therapeutic targets in immune-mediated and inflammatory skin diseases (29). In CSU, Qu et al. showed that miR-194 attenuates inflammatory responses and human dermal microvascular endothelial cell permeability by targeting THBS1 and suppressing TGF-β/SMAD signaling (27). Although miR-194 was not among the differentially expressed or candidate miRNAs in our dataset, these findings support the broader concept that miRNAs may contribute to vascular permeability and inflammatory regulation in urticaria. Lin et al. reported 16 differentially expressed miRNAs in patients with CSU and active hives, including increased miR-2355-3p, miR-4264, miR-2355-5p, miR-29c-5p, and miR-361-3p in patients with a positive CU index, suggesting potential biomarker relevance in autoimmune urticaria (28). No exact overlap was observed with our findings, however, both studies point to the miR-29 family, as Lin et al. identified miR-29c-5p, whereas our study found upregulation of miR-29c-3p and miR-29a-3p in CSU patients. This does not represent direct replication, given that these are different mature strands and/or family members, but it supports the possible involvement of miR-29-related regulatory pathways in chronic urticaria. The limited overlap between available CSU miRNA studies should be interpreted cautiously. Differences in patient selection, disease activity, biological material, profiling platforms, normalization strategies, and statistical thresholds may all influence miRNA discovery. Thus, the lack of exact overlap does not necessarily indicate contradictory findings, but rather highlights the need for larger, harmonized, and independently validated CSU miRNA studies.

Target interaction analysis revealed convergence of multiple dysregulated miRNAs on highly connected genes, including *MYC*, *GGA3*, *ACER2*, *CXCL8*, *PBX2*, and *SUOX*. Such convergence indicates coordinated post-transcriptional regulation affecting key cellular processes (35, 36). Network-based effects are consistent with models of chronic inflammation driven by distributed regulatory perturbations rather than single-gene alterations (31, 37). Among these targets, CXCL8 is of particular biological interest because of its established role in neutrophil recruitment and inflammatory amplification, processes that may contribute to systemic inflammatory activity in CSU. MYC and PBX2 may reflect broader transcriptional and immune-regulatory programs, whereas genes involved in cellular metabolism and stress responses may indicate adaptation to persistent inflammatory signaling. However, these target interactions are database-derived and should be interpreted as hypothesis-generating rather than direct experimental evidence.

Multi-group 50–50 MANOVA identified 61 significantly altered miRNAs, many overlapping with case–control results, indicating a dominant patient-versus-control signal. PCA confirmed clear separation between controls and patients but demonstrated substantial overlap among HSN, HSNA, and IA subgroups. These findings indicate that subgroup-level molecular differences are modest relative to global disease-associated changes. This is consistent with the biological relationship between CSU phenotypes, which share core mechanisms such as mast cell activation and mediator-driven inflammation (1). Clinically, this suggests that circulating miRNA profiles may capture a shared inflammatory state in CSU more strongly than they capture conventional phenotype labels. Therefore, the current clinical subdivision may not fully reflect the underlying molecular heterogeneity of the disease.

Pairwise subgroup analyses did not yield FDR-significant miRNAs. Nominally significant signals overlapped with the MANOVA-derived set, suggesting biologically relevant differences not fully captured by univariate models. Among these, miR-204-5p showed a consistent trend toward reduced expression in angioedema-associated subgroups and borderline statistical significance. Previous studies implicate miR-204 in immune regulation, endothelial function, and inflammatory signaling (38, 39), processes relevant to vascular permeability and angioedema in CSU. Although this finding did not reach robust statistical significance in subgroup comparisons, it provides a biologically plausible signal that warrants further investigation, particularly in studies specifically dedicated to examine angioedema-associated CSU.

Exploratory analyses suggested a potential association between miR-3158-3p and higher disease activity. This signal depended on incomplete and heterogeneous clinical metadata, limiting interpretability. These observations indicate that clinical covariates, including disease activity, may influence circulating miRNA profiles. Larger datasets with standardized and longitudinal phenotyping are required to validate such associations.

Independent enrichment analyses supported an immune-related context for observed miRNA alterations, including associations with epigenetic marks such as H3K36me3 across immune cell types. These findings are consistent with circulating miRNAs reflecting systemic immune activation states, immune cell composition shifts, or both (40, 41). This is relevant because circulating miRNAs may originate from several cellular sources, including immune cells, endothelial cells, platelets, and extracellular vesicles. Plasma miRNA alterations should be interpreted as systemic molecular signals rather than as direct readouts of a single pathogenic cell type.

Unsupervised clustering identified additional molecular structure beyond predefined clinical categories. Controls were clearly separated from CSU patients, whereas patient clusters contained mixed clinical labels. This indicates that molecular heterogeneity does not map directly onto current phenotype-based classification. These findings support endotype-based models of CSU, where underlying biological mechanisms are not fully captured by clinical presentation (42–44). This observation suggests that miRNA-based molecular profiling could eventually contribute to a more biologically informed classification of CSU. Such an approach may help identify patient subsets defined by inflammatory, autoimmune, endothelial, or treatment-response-related pathways rather than by symptoms alone.

Five miRNAs—miR-204-5p, miR-3158-3p, miR-4732-3p, miR-576-5p, and miR-877-5p—consistently emerged across analytical approaches. These miRNAs may represent candidate markers of disease-related regulatory processes. Their effects were modest and should be interpreted as exploratory. At the present stage, these miRNAs should not be considered validated diagnostic or prognostic biomarkers. Rather, they represent candidates for further validation in independent CSU cohorts and functional studies.

Available evidence for these miRNAs is limited and largely derived from non-CSU contexts. miR-204-5p has been linked to endothelial function, vascular homeostasis, and inflammatory signaling, including roles in pulmonary arterial hypertension and metabolic vascular dysfunction (38, 39). These processes are relevant to CSU, particularly in angioedema. miR-877-5p has been associated with cellular signaling in cancer and inflammatory models but lacks evidence in CSU or allergic disease (45–47). miR-3158-3p has been described mainly in cerebral malaria, with associations to disease severity and immune-related pathways (48, 49). miR-4732-3p has been linked to stress-related transcriptional responses in cardiovascular and oncological contexts (50–52). miR-576-5p has been associated with proliferation and signaling pathways such as MAPK and AKT in cancer models (53, 54). None of these miRNAs have established roles in CSU, supporting interpretation as exploratory markers.

These findings suggest that circulating miRNA profiles may reflect broader systemic immune or inflammatory states rather than discrete CSU phenotypes. The observed signals likely represent distributed regulatory changes across multiple pathways rather than specific disease-driving mechanisms. This interpretation is consistent with the complex and heterogeneous nature of CSU, in which mast cell activation, autoimmunity, inflammatory mediators, vascular permeability, and individual patient-specific factors may interact to produce overlapping clinical phenotypes.

From a clinical perspective, the potential relevance of these findings lies in the future use of circulating miRNAs as components of biomarker panels rather than as standalone markers. Such panels could potentially help characterize systemic inflammatory activity, identify molecular endotypes, estimate angioedema-associated risk, or predict response to targeted therapies. However, the present results are exploratory and are not yet suitable for direct clinical implementation. Before miRNAs can be used in clinical practice, candidate markers must be validated in larger independent cohorts, tested longitudinally, and linked to clinically meaningful outcomes such as disease activity, angioedema burden, treatment response, and remission.

Several limitations should be considered. The sample size was small, limiting statistical power, particularly for subgroup analyses. Functional interpretation relied on existing miRNA–target databases, which provide indirect and variable-quality evidence. Some identified miRNAs had low expression levels, increasing susceptibility to technical noise. Enrichment analyses did not yield strongly convergent pathway signals, suggesting diffuse biological effects rather than a single dominant mechanism. In addition, circulating miRNA profiles may be influenced by pre-analytical and biological factors, including sample handling, hemolysis, blood cell composition, comorbidities, medication use, and current disease activity. Among comorbidities, autoimmune and thyroid disorders, cardiovascular and metabolic diseases, and chronic inflammatory comorbidities may be particularly relevant, as they can independently affect systemic inflammation, endothelial function, immune activation, and circulating miRNA expression. These factors may complicate the distinction between CSU-specific miRNA signals and broader systemic or comorbidity-related effects and should be carefully controlled in future studies. Because the study was observational, causal relationships between miRNA expression and CSU pathogenesis cannot be inferred. Functional validation in cellular models, mast cell-based systems, endothelial models, and extracellular vesicle studies will be necessary to clarify whether the identified miRNAs contribute directly to disease mechanisms or primarily reflect systemic inflammatory activity.

## Conclusions

This study shows that circulating miRNA expression profiles differ between patients with CSU and healthy controls and are linked to pathways related to immune regulation, endothelial function, and stress-response signaling. While clinically defined subgroups demonstrated substantial overlap and limited molecular separation, multivariate and unsupervised analyses revealed molecular heterogeneity within the patient cohort.

Five miRNAs—miR-204-5p, miR-3158-3p, miR-4732-3p, miR-576-5p, and miR-877-5p—emerged across analyses as exploratory candidate biomarkers. These miRNAs may reflect disease-associated regulatory processes related to systemic inflammation, vascular permeability, and cellular stress responses. From a clinical perspective, circulating miRNAs may have future potential as components of biomarker panels for CSU endotyping, disease activity assessment, angioedema-associated mechanisms or risk, and treatment-response prediction. However, the present findings are exploratory and are not yet suitable for direct clinical application.

Validation is required in larger, independent, and well-characterized cohorts, preferably with longitudinal sampling, standardized disease activity assessment, treatment-response data, and orthogonal confirmation methods such as RT-qPCR to confirm their biological and clinical relevance.

## Data Availability

The data presented in the study are available here: https://doi.org/10.48510/FK2/XG4FU2.
